# Whole‐brain DTI parameters associated with tau protein and hippocampal volume in Alzheimer's disease

**DOI:** 10.1002/brb3.2863

**Published:** 2023-01-04

**Authors:** Thamires Naela Cardoso Magalhães, Raphael Fernandes Casseb, Christian Luiz Baptista Gerbelli, Luciana Ramalho Pimentel‐Siva, Mateus Henrique Nogueira, Camila Vieira Ligo Teixeira, Ana Flávia Mac Knight Carletti, Thiago Junqueira Ribeiro de Rezende, Helena Passarelli Giroud Joaquim, Leda Leme Talib, Orestes Vicente Forlenza, Fernando Cendes, Marcio Luiz Figueredo Balthazar

**Affiliations:** ^1^ Department of Neurology and Neuroimaging Laboratory School of Medical Sciences University of Campinas (UNICAMP) Campinas Brazil; ^2^ Laboratory of Neuroscience (LIM‐27) Department and Institute of Psychiatry University of Sao Paulo (USP) São Paulo Brazil; ^3^ Seaman Family MR Research Center University of Calgary Calgary Canada; ^4^ Brazilian Institute of Neuroscience and Neurotechnology São Paulo Brazil; ^5^ National Institute on Aging, National Institute of Health Baltimore Maryland USA

**Keywords:** Alzheimer's disease pathology, biomarkers, CSF proteins, DTI measures, white matter damage

## Abstract

The causes of the neurodegenerative processes in Alzheimer's disease (AD) are not completely known. Recent studies have shown that white matter (WM) damage could be more severe and widespread than whole‐brain cortical atrophy and that such damage may appear even before the damage to the gray matter (GM). In AD, Amyloid‐beta (Aβ_42_) and tau proteins could directly affect WM, spreading across brain networks. Since hippocampal atrophy is common in the early phase of disease, it is reasonable to expect that hippocampal volume (HV) might be also related to WM integrity. Our study aimed to evaluate the integrity of the whole‐brain WM, through diffusion tensor imaging (DTI) parameters, in mild AD and amnestic mild cognitive impairment (aMCI) due to AD (with Aβ_42_ alteration in cerebrospinal fluid [CSF]) in relation to controls; and possible correlations between those measures and the CSF levels of Aβ_42_, phosphorylated tau protein (p‐Tau) and total tau (t‐Tau). We found a widespread WM alteration in the groups, and we also observed correlations between p‐Tau and t‐Tau with tracts directly linked to mesial temporal lobe (MTL) structures (fornix and hippocampal cingulum). However, linear regressions showed that the HV better explained the variation found in the DTI measures (with weak to moderate effect sizes, explaining from 9% to 31%) than did CSF proteins. In conclusion, we found widespread alterations in WM integrity, particularly in regions commonly affected by the disease in our group of early‐stage disease and patients with Alzheimer's disease. Nonetheless, in the statistical models, the HV better predicted the integrity of the MTL tracts than the biomarkers in CSF.

## Graphical abstract

It is known that significant neuropathological changes occur in the preclinical phase in Alzheimer's disease (AD), requiring the identification of metrics that can detect such early changes. While cerebrospinal fluid (CSF) measures of amyloid and tau proteins are generally accepted as biomarkers of AD pathology, neuroimaging measures used to indicate white matter alterations throughout the brain remain less widely endorsed as candidate biomarkers.

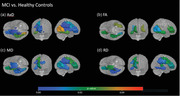

## 1 INTRODUCTION

Alzheimer's disease (AD) is the most common neurodegenerative disease affecting the elderly population. According to United Nations, the number of older people in less developed regions has grown considerably, and projections indicate that 1.7 billion people aged 60 years or over will live in these regions by 2050 (Burla et al., 2013; Reitz et al., 2011). According to World Health Organization (WHO, 2022), the global prevalence of dementia has been estimated to be as high as 55 million and it is predicted to double until 2040 (United Nations, Department of Economic & Social Affairs, Population Division, 2015).

The causes of the AD neurodegenerative process are not completely known and involve different mechanisms in the pathogenesis. Although remarkable pathological characteristics have already been identified and used as biomarkers, especially regarding cerebrospinal fluid (CSF) proteins – (Amyloid‐beta [Aβ_42_] peptide, phosphorylated tau [p‐Tau] protein, and total tau [t‐Tau]) (WHO, 2022), other characteristics are yet to be determined as useful biomarkers. Some of these biomarkers can be accessed through neuroimaging analysis.

AD is classically considered a gray matter (GM) disease that starts in mesial temporal lobe (MTL) structures. The transentorhinal cortex is one of the first affected areas and then the changes spread to limbic and neocortical regions. Hippocampal atrophy is also a very distinctive biomarker of the disease, but not exclusive (Braak et al., 1999).

Recent studies have shown that white matter (WM) damage could be more severe and widespread than whole‐brain cortical atrophy (Falgàs et al., 2019). Some authors state that WM damage can be detected before the beginning of cortical atrophy and overt dementia in mild cognitive impairment (MCI), and even in cognitive impaired elders (Falgàs et al., 2019; Stone et al., 2021) or even in asymptomatic stages (Amlien & Fjell, 2014).

Although the WM alterations in the AD spectrum has been reported, little is known regarding their etiology and relationship with Amyloid and tau proteins. One of the hypotheses that relates damage to WM is that the propagation pattern of misfolded proteins via interconnected neural networks in a prion‐like mechanism could directly damage WM (Van Schependom et al., 2018). The Aβ peptide and/or tau protein might spread across brain networks through WM tracts that connect remote regions of those networks (Chen et al., 2018; Colin et al., 2020). This proliferation of abnormal proteins could then lead to the progressive neuronal death.

Studies that approach the diffusion tensor imaging (DTI) analysis and its WM integrity parameters showed several changes in AD (Vaquer‐Alicea & Diamond, 2019). These diffusivity parameters indicate axonal injuries and are increased in the disease. Radial diffusivity (RD) is more sensitive to changes in axon diameter and density: high RD values indicate a smaller thickness of the myelin sheath. The axial diffusivity (AxD) represents the maximum direction of diffusion and gives a primary estimate of the direction of the fiber (Falgàs et al., 2019; Stone et al., 2021). On the other hand, for the fractional anisotropy (FA) measure, the higher this parameter, the more preserved the tract is. And in AD, FA is usually reduced (Falgàs et al., 2019; Stone et al., 2021; Vaquer‐Alicea & Diamond, 2019).

The DTI parameters could be related to CSF proteins, explaining the spread pattern and alterations as mentioned above (Kantarci et al., 2017; Vaquer‐Alicea & Diamond, 2019). However, since hippocampal volumetric atrophy is observed in patients with amnestic mild cognitive impairment (aMCI) and in the pathological process of the disease, it is reasonable to expect that those alterations might be influencing these interactions between CSF protein and WM (Kantarci et al., 2017; Knopman et al., 2018).

As the association between AD biomarkers and WM integrity throughout the brain remains unclear, especially regarding aMCI in the Alzheimer´s continuum (with Aβ_42_ alteration—lower levels in the CSF), in this study, we aimed to evaluate (1) differences in whole brain‐WM integrity (DTI parameters), in mild AD and aMCI due to AD in relation to controls; (2) possible correlations between CSF measures of Aβ_42_, p‐Tau and t‐Tau and DTI parameters in aMCI due to AD and mild AD; and (3) if we observe alterations in regions characteristically related to AD, that is, regions belonging to the MTL, we intend to analyze the possible effect of the hippocampus volume (HV), since as mentioned earlier, AD is classically considered a GM disease, and neurodegenerative changes within the hippocampus may be resulting in the degeneration of associated WM fibers.

## 2 METHODS

### 2.1 Participants

One hundred and eighty‐three individuals were enrolled in this study: 48 aMCI, 30 patients with very mild or mild AD dementia and 105 normal controls. All participants underwent neuropsychological evaluation, recently described in Magalhães et al. (2021), performed by an experienced neuropsychologist, and MRI exam (Anderson et al., 2012).

The diagnosis of probable dementia due to AD fulfilled the criteria defined by the National Institute of Aging and Alzheimer's Association (NIA/AA) (Magalhães et al., 2021) and all AD patients had a Clinical Dementia Rating (CDR) (McKhann et al., 2011) score of 0.5 or 1. Patients with aMCI were diagnosed using the core criteria of the NIA/AA for MCI—CDR score of 0.5(Morris, 1993)—and had pathophysiological evidence of AD (AD *continuum*), characterized by low CSF Aβ_42_ (<540pg/ml), according to manufacturer's recommendations (amyloid‐β INNOTEST® kit—Fujirebio).

Controls were identified as cognitively normal: they did not exhibit any neurological or psychiatric disorders or require psychoactive medication. They demonstrated normal Mini Mental State Examination scores, considering age and education in agreement with the study of Brucki et al. (2003) (Albert et al., 2011), and their structural images were characterized by the absence of abnormalities. The control subjects had neither memory complaints nor neurological deficits, and they presented a CDR score of zero.

Exclusion criteria for all subjects included the following: other neurological or psychiatric diseases or having suffered a head trauma that resulted in a loss of consciousness, drug or alcohol addiction, use of sedative drugs 24 h before neuropsychological evaluation, prior chronic exposure to neurotoxic substances, Fazekas score >1, and a Hachinski ischemic score >4 (Brucki et al., 2003).

Pre‐diagnostic procedures also included laboratory tests including Vitamin B12, folate, syphilis serology, and thyroid hormones.

Regarding the influence of vascular effects, all individuals of this study were normal for age concerning WM hyperintensities on MRI, presenting Fazekas scale 0 or 1.

### 2.2 CSF sample collection and quantification of biomarkers

Patients with aMCI and mild AD were prescribed for CSF collection by a lumbar puncture. For ethical reasons, we did not collect CSF from our healthy controls group. The CSF collection and the quantification of proteins (Aβ_42_, t‐Tau and p‐Tau) are also described by Magalhães et al. (2021) (Hachinski et al., 2006; Knopman et al., 2018). Measurements of Aβ_42_, t‐Tau and p‐Tau were obtained using amyloid‐β INNOTEST® kits (Albert et al., 2011; Amlien & Fjell, 2014; Anderson et al., 2012; Asai et al., 2015; Braak et al., 1999; Brucki et al., 2003; Burla et al., 2013; Chen et al., 2018; Colin et al., 2020; Falgàs et al., 2019; Faria et al., 2010; Fiock et al., 2020; Franzmeier et al., 2020; Gold et al., 2014; Hachinski et al., 2006; Jacobs et al., 2018; Kantarci et al., 2017; Karran et al., 2011; Knopman et al., 2018; Magalhaes et al., 2018; Magalhães et al., 2021; McKhann et al., 2011; Michel et al., 2014; Morris, 1993; Pontecorvo et al., 2019; Racine et al., 2014; Reitz et al., 2011; Rezende et al., 2019; Sabuncu et al., 2010; Sanchez et al., 2021; Soldan et al., 2020; Stone et al., 2021; Takeda et al., 2015; Tang et al., 2014; United Nations, Department of Economic & Social Affairs, Population Division, 2015; Van Schependom et al., 2018; Vaquer‐Alicea & Diamond, 2019; Vogel et al., 2020; Woods et al., 1998; WHO, 2022; Zhuang et al., 2006), h‐TAU INNOTEST® Ag, and INNOTEST® Phospho‐tau (181P) (Fujirebio), respectively.

### 2.3 MRI acquisition

All magnetic resonance images (MRI) were acquired on a Philips® Achieva 3.0T scanner within a week after the neuropsychological test. The following protocol was applied to each subject: (a) for the hippocampal volume analyses: sagittal high‐resolution T1‐weighted (isotropic voxels of 1 × 1 × 1 mm^3^, TR/TE = 7/3.2 ms, FOV = 240 × 240 mm, 180 slices); (b) for the WM microstructural analysis, a standard DTI protocol was performed (acquired voxel size = 2 × 2 × 2 mm^3^ reconstructed with 1 × 1 × 2 mm^3^, no gap, TR = 8.5 s, TE = 61 ms, 32 diffusion directions with *b* = 1000 s/mm^2^, acquisition matrix = 128 × 128 reconstructed to 256 × 256).

To classify subjects according to Fazekas scale, we acquired coronal and axial fluid attenuated inversion recovery (FLAIR) T2‐weighted images, anatomically aligned to the hippocampus (reconstructed voxel size = 0.45 × 0.45 × 4.00 mm^3^, TR/TE/TI = 12,000/140/2850 ms, FOV = 220 × 206 mm, gap = 1 mm).

#### 2.3.1 DTI processing and automated segmentation

To assess the WM microstructural abnormalities, we employed the MRICloud (www.MRICloud.org), a recently developed web‐based tool to perform automated segmentation and quantification of multiple MRI modalities. MriCloud provides a platform to characterize anatomy (using T1 high resolution weighted images for volumetric analysis), WM (using diffusion tensor images DTI), and resting state functional connectivity, based on the structure‐based analysis (Magalhaes et al., 2018). In terms of reproducibility, MRICloud showed high indexes, especially to the DTI pipeline, revealing that such methodology is a reasonably stable tool (Mori et al., 2016). Indeed, anatomical heterogeneity can insert errors into the registration between each patient images and the parcellation atlases. To correct this problem, MRICloud uses a database of atlases obtained from brains with heterogeneous anatomy. Therefore, some atlases would be anatomically closer to the patient than other atlases, providing a high level of parcellation accuracy (Rezende et al., 2019). Lastly, to the best of our knowledge, MRICloud is the only automated pipeline available for whole‐brain DTI providing almost 200 anatomical structures.

This technique is based on the probabilistic determination of the water molecule diffusion in the tissue, helping to quantify the integrity of the brain tracts (Tang et al., 2014). To extract the main diffusion indices, we used an automated segmentation based on multiple labeled atlases approach, which is a more detailed and refined examination that can assess thinner and smaller tracts. It enables the analysis of alterations that may be more subtle in early stages of the disease.

To accomplish that, initially the images were corrected for eddy currents and co‐registered using a 12‐parameter affine transform (Faria et al., 2010; Woods et al., 1998) to remove subject motion. The images were skull‐stripped using the *b* = 0 image by intensity threshold approach, which is implemented at the RoiEditor software (Li, X.; Jiang, H.; Yue, L.; and Mori, S.; Johns Hopkins University, www.MriStudio.orgorwww.kennedykrieger.org).

The DTI‐maps are calculated using a multivariate linear fitting as implemented in the DTIStudio software (Jiang, H. and Mori, S., Johns Hopkins University, Kennedy Krieger Institute). Before performing the parcellation, which employs the diffeomorphic likelihood fusion algorithm (DLFA) algorithm, the images are registered to the atlases using the multi‐contrast large deformation diffeomorphic metric mapping (LDDMM) algorithm (Zhuang et al., 2006).

We then obtained the main diffusion parameters (FA, mean diffusivity [MD], RD, and AxD) and the volume for each label. Analyzing more than one measure allows us to consistently assess whether in fact there is any WM alteration. All analyses are performed in native space.

##### Selection of WM tracts

The processing described above provided FA, AxD, MD, and RD metrics for 64 WM tracts (described in Table S1). Thus, to avoid overfitting and reduce the number of comparisons, we performed a principal component analysis (PCA) to evaluate the regions of interest (ROIs) that have contributed the most to WM variability in our sample. Those results guided the selection of variables to perform group comparisons in a data‐driven approach.

We performed separated PCAs on WM tracts data, one for each DTI parameter including all participants (controls and patients), as mentioned above, and the purpose of the analysis is to evaluate the ROIs that have contributed the most to WM variability, and multicollinearity was tested. Thus, we could preserve statistical power while avoiding bias by selecting ROIs that would be associated to a specific group already a priori. Both Keiser's and Catell's criteria were applied to extract factors.

After extraction, components were rotated using the non‐orthogonal direct *oblimin* method. Sample size and model adequacy were evaluated using Barlett's test of sphericity and the Keiser–Meyer–Olkin (KMO) measure. To ensure inclusion of relevant ROIs, we selected within the main components those ROIs with loadings >0.7 (meaning a strong relationship contributing with at least 64% of variability contribution in the component) to perform hypothesis testing (described in Table S2).

All PCAs showed a KMO >0.883, suggesting an appropriate adjustment to the data. Analysis of both Kaiser's and Catell's criteria suggested that the variability was concentrated on the first two components. We then repeated the analysis extracting only a fixed number of two components explaining a total variance of 40% for FA, 47% for AxD, 55% for MD, and 54% RD. We then selected those WM ROIs with loading factors >0.7 in the subsequent analysis.

#### 2.3.2 Hippocampal Analyses—FreeSurfer

To obtain the HV values of the individuals we used FreeSurfer software v.5.3 (Soldan et al., 2020) (https://surfer.nmr.mgh.harvard.edu). In this processing, cortical surface reconstruction is performed and anatomical segmentation of MRI brain scans.

We processed all high‐resolution T1‐weighted MR volumetric images through the default FreeSurfer processing stages to perform non‐linear registration (warping) from the original space to the MNI305 space (standard space), cortical and subcortical segmentations, and cortical thickness measurements. We visually confirmed the accuracy of warping the T1‐weighted MR volumetric images to the standard space. Macroscopic artifacts affected none of the T1‐weighted volumes of the participants.

For all analyses, a Gaussian filter with a 10‐mm full width at half maximum was used for smoothing the surface. The volume of individual structure was computed from labeled voxels and normalized to the total intracranial volume.

### 2.4 Statistical analyses

All statistical analyses were carried out in SPSS package (version 22, SPSS Inc., Chicago, IL, USA). We performed a univariate analysis of covariance (ANCOVA) to compare the neuropsychological test and demographic data between the groups using the Bonferroni post hoc test, setting *p* < .05 as significant. Chi‐square test was used for categorical analyses (sex). Because age and education were significantly different among groups, we included these variables as nuisance covariates in the analyses.

T‐test was used to compare CSF proteins levels between mild AD and aMCI. To compare the DTI measures between the groups, we performed a multivariate analysis of covariance with the Bonferroni post hoc test. To verify the association between DTI data and CSF biomarkers, we performed partial correlations, including age and education as covariates.

We then followed up the significant partial correlations with linear regressions to better evaluate the effect of HV on the relationship between the DTI measures and CSF proteins in aMCI and mild AD groups. We build hierarchical regressions models separated by patient groups (aMCI and mild AD) with each DTI region as independent variable, right HV in the first block as covariate and CSF proteins in the second block, using the enter method. The model adjustment was evaluated by residual analysis. No serious violations were found.

## 3 RESULTS

### 3.1 Neuropsychological and demographic data

There were significant differences in age and education, but not in sex, between mild AD and aMCI patients compared to controls. As expected, AD and aMCI patients performed worse than controls in the neuropsychological tests as shown in Table 1.

**TABLE 1:** Descriptive and group comparison of demographic, main neuropsychological data, and biomarkers, significance adjusted for age and education (years)

	Controls	aMCI		Mild AD		
	(*n* = 105)	(*n* = 48)	*p*‐Value (aMCI × controls)	(*n* = 30)	*p*‐Value (mild AD × controls)	*p*‐Value (mild AD × aMCI)
Age	60 ± 7.29	68 ± 7.13	0.067	73 ± 7.56 ^a^, *** ^,^ ^b^, *	0.00	0.046
	66 (10)	66 (11)		74 (10)		
Education (years)	11.66 ± 5.05	9.39 ± 5.78 ^a^, *	0.048	6.5 ± 4.96 ^a^, ***	0.00	0.059
	14 (5)	11 (13)		5 (5)		
CDR	0	0.5 ± 0 ^a^, ***	0.00	0.87 ± 0.21 ^a^, *** ^,^ ^b^, ***	0.00	0.00
Pfeffer	0.53 ± 0.80	3.33 ± 3.04 ^a^, **	0.033	12.97 ± 5.17 ^a^, *** ^,^ ^b^, ***	0.00	0.00
	0 (1)	1 (4)		10 (2)		
MMSE	28.42 ± 1.49	25.59 ± 2.88 ^a^, ***	0.00	20.1 ± 5.92 ^a^, *** ^,^ ^b^, ***	0.00	0.00
	29 (3)	26 (4)		23 (5)		
RAVLT	45.16 ± 8.44	30.54 ± 8.03 ^a^, ***	0.00	21.77 ± 12.33 ^a^, *** ^,^ ^b^, ***	0.00	0.00
	40.5 (15)	30 (7)		23 (8)		
A7	8.95 ± 2.81	3.78 ± 2.24 ^a^, ***	0.00	1.03 ± 1.58 ^a^, *** ^,^ ^b^, ***	0.00	0.00
	7.5 (4)	3 (3)		0 (2)		
CSF biomarkers (pg/ml)						
t‐Tau	NA	102.79 ± 66.55	NA	142.34 ± 79.32 ^b^, *	NA	0.03
		78.68 (65.58)		119.27 (119.7)		
p‐Tau	NA	45.75 ± 23.66	NA	58.97 ± 36.80	NA	0.07
		41.06 (27.8)		46.67 (30.4)		
Aβ_42_	NA	375.44 ± 105.81	NA	333.87 ± 113.86	NA	0.18
		391.94 (145.69)		348.34 (185.65)		
Aβ_42_ /t‐Tau	NA	5.06 ± 2.96	NA	3.22 ± 2.4 ^b^, **	NA	0.025
		4.64 (4.23)		2.3 (2.74)		
Aβ_42_ /p‐Tau	NA	11.33 ± 8.5	NA	7.95 ± 7.19	NA	0.23
		8.43 (8.91)		5.74 (5.63)		
Hippocampal volume (mm^3^)						
Left hippocampus (10^‐3^)	2.86 ± 0.44	2.53 ± 0.55 ^a^, ***	0.005	2.09 ± 0.34 ^a^, *** ^,^ ^b^, ***	0.00	0.002
	2.96 (0.1)	2.61 (0.1)		0.2 (0.04)		
Right hippocampus (10^‐3^)	3.05 ± 0.49	2.71 ± 0.56 ^a^, ***	0.009	2.1 ± 0.42 ^a^, *** ^,^ ^b^, ***	0.00	0.003
	3.17 (0.07)	2.87 (0.12)		2.02 (0.03)		

*Note*: Mean ± standard deviation/Median (Interquartile range). Statistical analysis: ANCOVA with Bonferroni post hoc test. Pfeffer: Pfeffer Functional Activities Questionnaire.

Abbreviations: Aβ, amyloid β peptide; AD, Alzheimer's disease; CDR, Clinical Dementia Rating; MMSE, Mini Mental State Examination; NA, not available; p‐Tau, phosphorylated‐tau; RAVLT, Rey Auditory Verbal Learning Test; t‐Tau, total tau protein; A7, Rey Auditory Verbal Learning Test‐delayed recall.

^a^ Different from controls.

^b^ Different from aMCI.

* *p* < .05

** *p* < .01

*** *p* < .001.

### 3.2 CSF biomarker levels

Comparing CSF biomarker levels between mild AD and aMCI in the AD continuum, we observed that, regarding t‐Tau protein levels, the mild AD group showed higher levels than the aMCI group and aMCI Aβ_42_/t‐Tau levels were higher than the mild AD group, as shown Table 1.

As expected, although not statistically significant, the AD group showed higher p‐Tau and t‐Tau and smaller Aβ_42_ values than aMCI.

### 3.3 Hippocampal volumes

As expected, aMCI and mild AD presented smaller HVs in relation to controls, and AD presented smaller HVs in relation to aMCI (Table 1).

### 3.4 Group comparison of WM integrity

**FIGURE 1:**
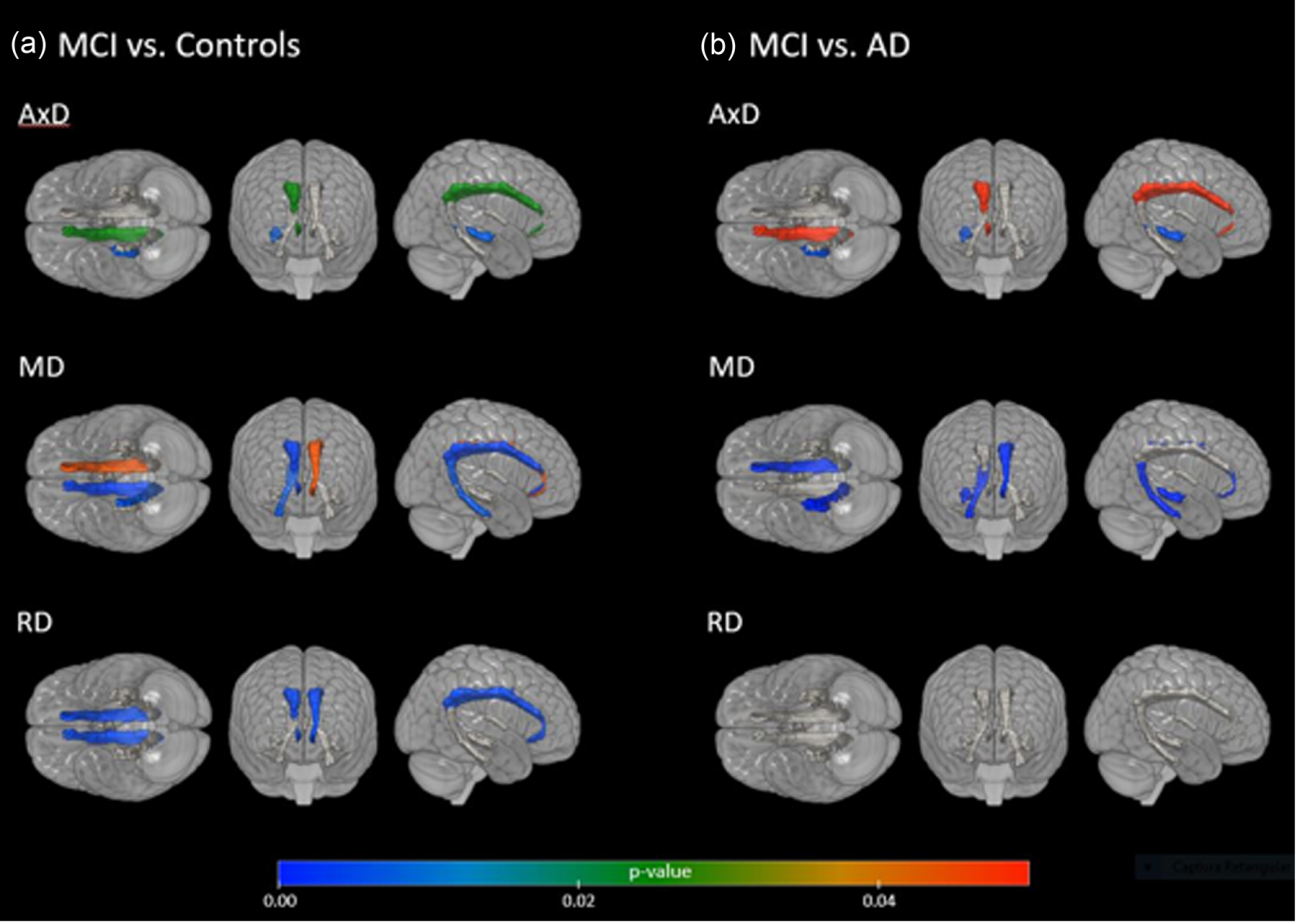
Significant differences in diffusion tensor imaging (DTI) metrics (axial diffusivity [AxD], mean diffusivity [MD], and radial diffusivity [RD]) in main regions: fornix, cingulum, and hippocampal‐cingulum between amnestic mild cognitive impairment (aMCI) versus healthy controls and aMCI versus mild Alzheimer's disease (AD) patients. According to the figure, (A) it is possible to observe that the aMCI group, when compared with the control group, presents differences in the measures that represent damage in the white matter (WM). That is, the WM of the aMCI group is less integrated than when compared to the control group; (b) the WM of the mild AD group is less integrated than when compared to the aMCI.

**FIGURE 2:**
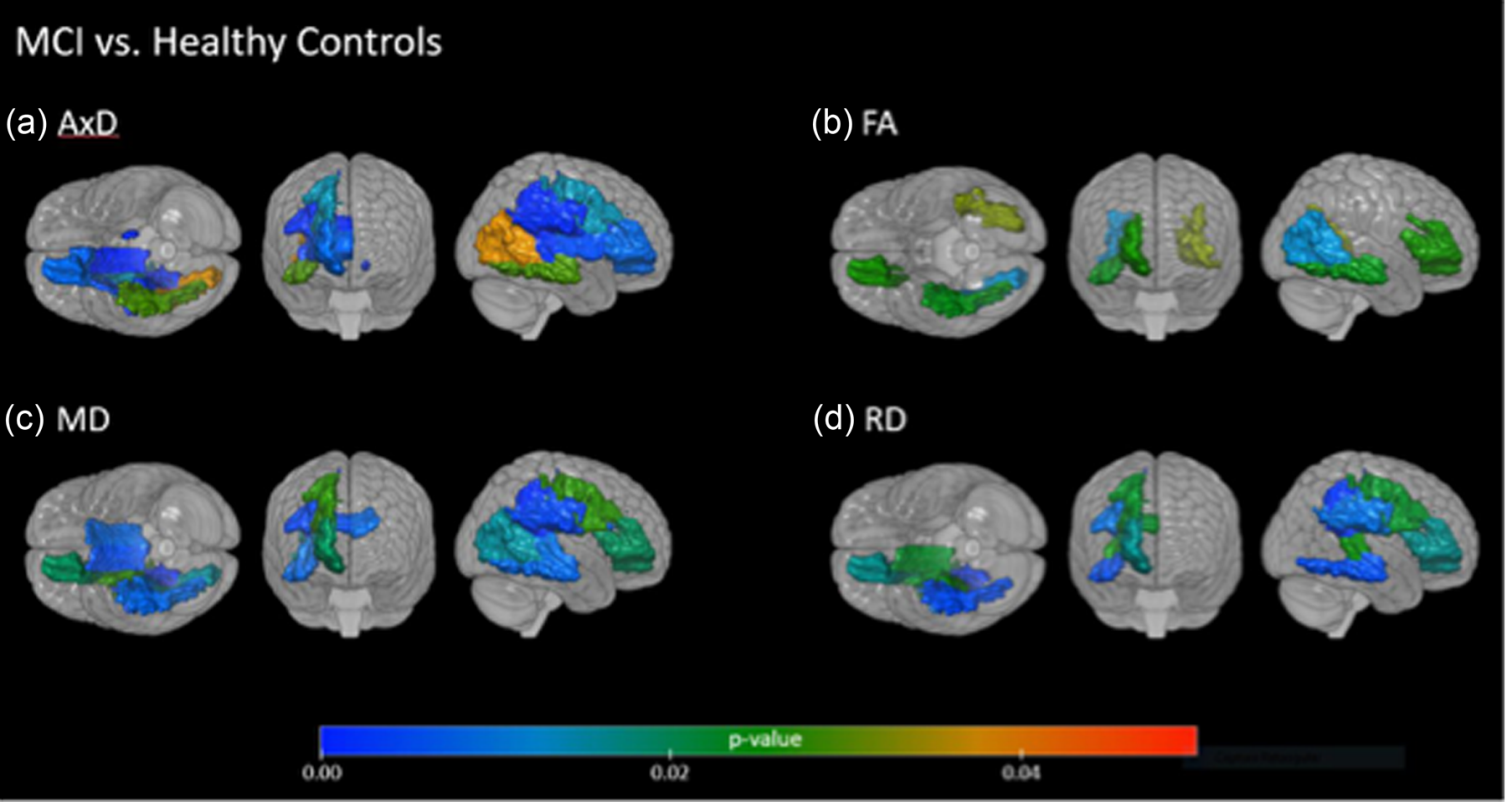
Significant differences in diffusion tensor imaging (DTI) metrics comparing amnestic mild cognitive impairment (aMCI) and healthy controls in other brain regions. (a) For the axial diffusivity (AxD): the aMCI group is less integrated than the control group in tracts such as corona radiata and corpus callosum; (b) for the fractional anisotropy (FA): the aMCI group is less integrated than the control group in tracts such as corona radiata and posterior thalamic radiation; (c) for the mean diffusivity (MD): the aMCI group is less integrated than the control group in tracts such as corona radiata, corpus callosum, superior longitudinal fasciculus and inferior fronto‐occipital fasciculus; (d) for the radial diffusivity (RD): the aMCI group is less integrated than the control group in tracts such as corona radiata, corpus callosum, and superior fronto‐occipital fasciculus.

**FIGURE 3:**
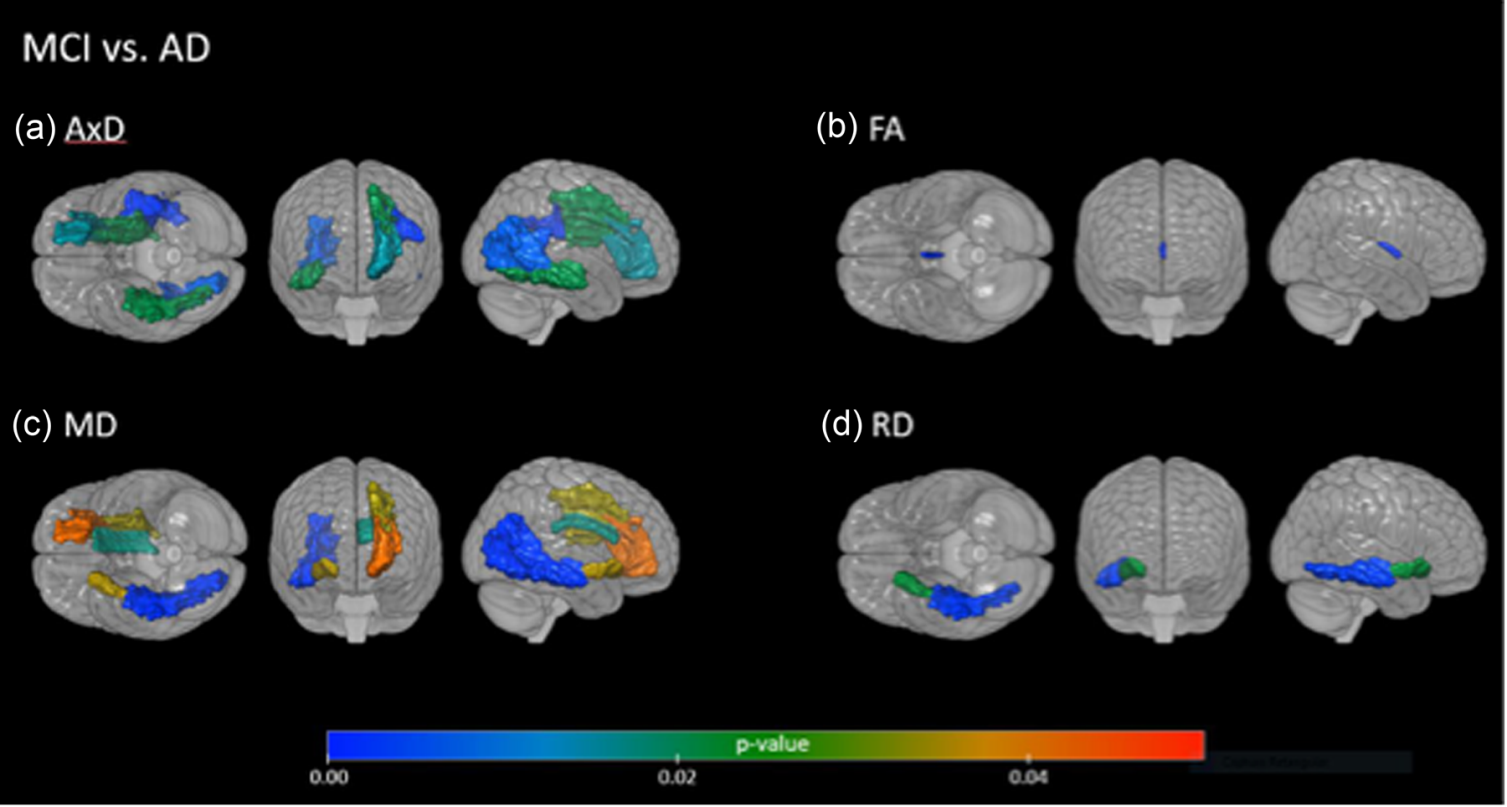
Significant differences in diffusion tensor imaging (DTI) metrics comparing amnestic mild cognitive impairment (aMCI) and mild Alzheimer's disease (AD) patients in other brain regions. (a) For the axial diffusivity (AxD): the mild AD group is less integrated than when compared to the aMCI group in tracts such as superior longitudinal fasciculus, corona radiata, and posterior thalamic radiation; (b) for the fractional anisotropy (FA): the mild AD group is less integrated than when compared to the aMCI group in the right fornix tract; (c) for the mean diffusivity (MD): the mild AD group is less integrated than when compared to the aMCI group in tracts such as inferior fronto‐occipital fasciculus, corona radiata, corpus callosum, and posterior thalamic radiation; (d) for the radial diffusivity (RD): the mild AD group is less integrated than when compared to the aMCI group in tracts such as inferior fronto‐occipital fasciculus and sagittal stratum.

After the statistical analysis with the PCA, the regions that best represented the statistical model were compared between the groups. As indicated by the PCA, the following regions contributed the most for the variability observed in patients: for FA: right fornix, left and right genu of corpus callosum, left and right posterior thalamic radiation; for AD: left and right body of corpus callosum, right cingulum, corona radiata regions; for RD: right and left cingulum, corona radiata regions, right superior longitudinal fasciculus; and for MD: left and right genu of corpus callosum, right cingulum‐hippocampal, corona radiata regions, as illustrated in Figures 1, 2, 3.

We observed alterations in MTL WM tracts including the hippocampal‐cingulum, fornix, and cingulum between aMCI versus controls and aMCI versus mild AD, as represented in Figure 1.

We also observed that the AxD, RD, and MD measurements showed more differences between the aMCI and controls groups than the FA measure.

Overall, we noticed that mild AD and aMCI groups presented higher values of AxD, MD, and RD, as well as lower FA values than the control group, and the results are described in Table S3.

We also found changes in other regions than MTL tracts between aMCI patients versus controls (Figure 2), and between patients with aMCI and mild AD (Figure 3), such as tracts of the commissural fibers, corona radiata, sagittal striatum, and thalamic radiation. Emphasizing that the measures that imply lesions (AxD, RD, and MD) are increased in patients than in comparison with controls.

### 3.5 Correlations and regressions between CSF proteins and DTI measures

**FIGURE 4:**
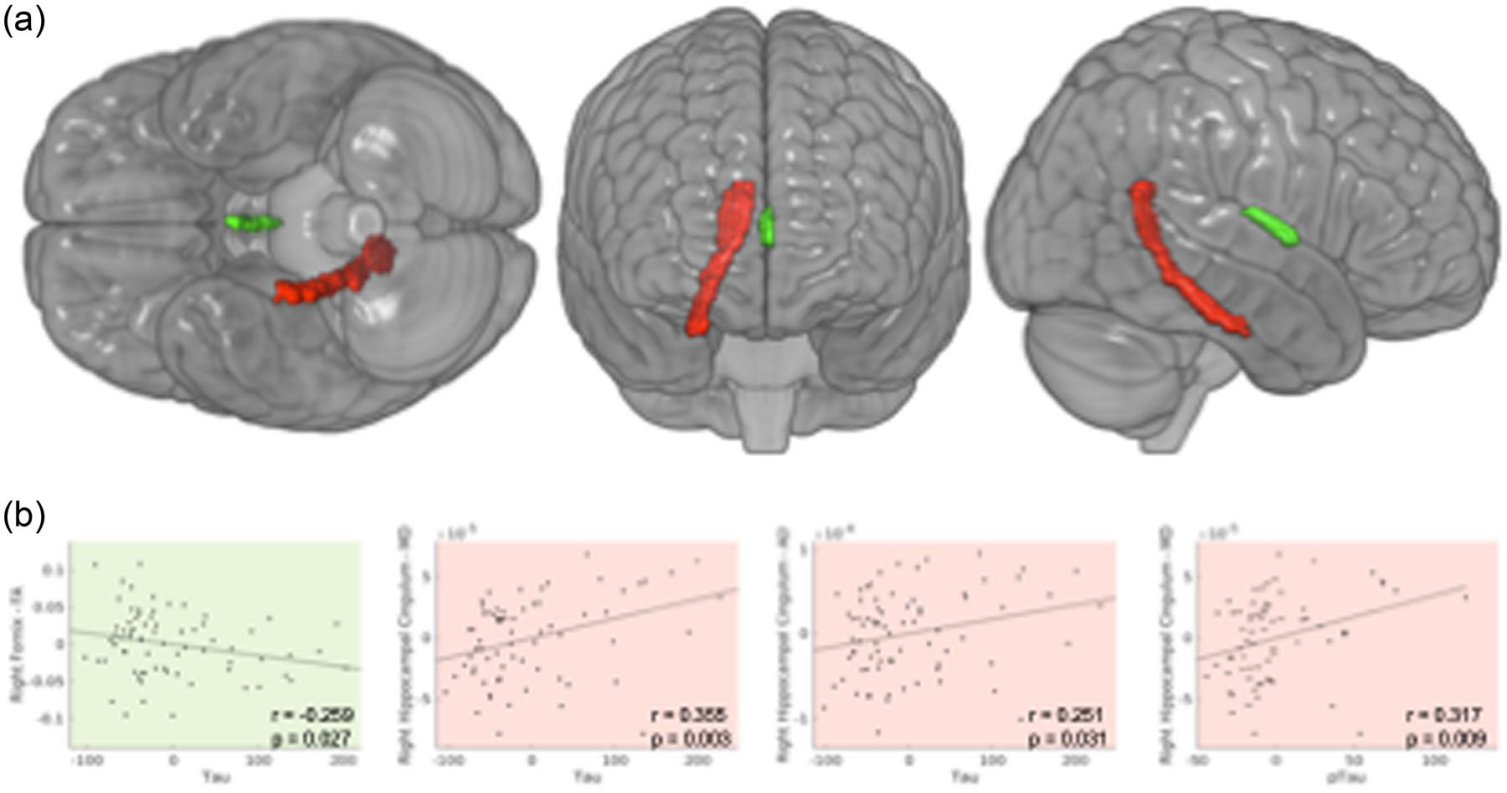
Partial correlations (r) between white matter diffusion tensor imaging (DTI) parameters and cerebrospinal fluid levels of tau and phosphorylated tau (p‐Tau) in patients (amnestic mild cognitive impairment and Alzheimer's disease patients). (a) Anatomical representation of the right hippocampal cingulum (red) and the right fornix (green) and (b) graphs of significant correlations. The background color indicates the anatomical region correlated with the DTI parameters.

When considering aMCI and mild AD groups together, we found significant partial correlations, considering age and sex as covariables, between WM tracts and tau and p‐Tau, but not with Aβ_42_. Significant results were found as follows: FA of right fornix (*r* = −0.259; *p* = .027), MD (*r* = 0.355; *p* = .003), and AxD (*r* = 0.251; *p* = .03) of right cingulum hippocampal. Concerning p‐Tau, we found significant correlation with AxD of right cingulum hippocampal (*r* = 0.317, *p* = .009), as shown in Figure 4.

In summary, we observed that the higher the CSF level of t‐Tau, the lower the FA measurement values in the region of the right fornix and the higher the values of the MD and AxD measurements in right cingulum hippocampal region. And as for p‐Tau values, the higher the levels in the CSF, the greater the MD measure in right cingulum hippocampal.

Next, we evaluated if those significant correlations between tau and p‐Tau with WM microstructure remained after controlling for hippocampal volumes. If so, we could speculate that those associations were independent of hippocampal integrity. However, no significant correlations were seen after controlling for HV.

Due to those results, to further test the influence of the HV in the model, we performed linear regressions and observed that the HV better explained the variation found in the DTI measures (with weak to moderate effect sizes, explaining from 9% to 31%) than did CSF proteins.

Only HV remained as significant predictor of DTI measures in each model, as follows: for aMCI group, right fornix FA (*β* = 36.13(95% IC 7.14–65.12), *R* ^2^ = 0.21, *p* = .016; for t‐Tau, p‐Tau, and Aβ_42_ all *p* > .69); right hippocampal‐cingulum AD (*β* = .45(95% IC 0.07–0.01), *R* ^2^ = 0.15, *p* = .004; for t‐Tau, p‐Tau, and Aβ_42_ all *p* > .3); right hippocampal‐cingulum MD (*β* = .03 (95% IC 0.05–0.01), *R* ^2^ = 0.16, *p* = .006); for t‐Tau, p‐Tau, and Aβ_42_ all *p* > .13. For the mild AD group, right fornix FA (*β* = 29.89 (95% IC 14.01–73.8), *R* ^2^ = 0.09, *p* = .17); for t‐Tau, p‐Tau, and Aβ_42_ all *p* > .51; right hippocampal‐cingulum AD (*β* = .07 (95% IC 0.11–0.02), *R* ^2^ = 0.31, *p* = .005); for t‐Tau, p‐Tau, and Aβ_42_ all *p* > .16; right hippocampal‐cingulum MD (*β* = .03 (95% IC 0.06–0.003), *R* ^2^ = 0.21, *p* = .03); for t‐Tau, p‐Tau, and Aβ_42_ all *p* > .44.

## 4 DISCUSSION

In the present study, we first evaluated whole‐brain differences in WM microstructural integrity in the AD continuum (aMCI with altered Aβ_42_ levels in CSF and mild AD patients) and controls. Our results showed a spread of WM alterations. We observed differences in several different regions, not only in the MTL. The integrity of tracts of the corpus callosum, corona radiata and thalamus are different between groups according to diffusivity measurements, indicating that the aMCI and mild AD groups presented more WM alterations than the control group.

We also verified if tau and Aβ_42_ could be related with WM microstructure in different tracts throughout the brain, not only in regions that are classically involved with AD. We hypothesized that significant correlations with tracts anatomically and functionally far from more well‐established atrophic regions, that is, regions involving the MTL, would support a more direct effect of the pathological AD proteins on WM (Sabuncu et al., 2010; Soldan et al., 2020). By the other hand, alterations found close to regions like hippocampus would imply different pathological mechanisms, like Wallerian degeneration. We did not find significant results regarding Aβ_42_ levels and DTI parameters, but we observed correlations of t‐Tau and p‐Tau protein with measures of FA, AxD, and MD, in the right fornix and right hippocampal‐cingulum tracts (Figure 4). However, through regression analysis, only HV remained as significant explanatory variable of CSF biomarkers.

The pattern of WM disruption in early stages of AD could initially take place in limbic and commissural tracts and, later, may progress to the projection and association fibers (Falgàs et al., 2019). Recent studies have demonstrated the mechanism through which tau pathology initially progresses from distal axons to proximal dendrites, leading to synaptic disconnection of late myelination fibers (Colin et al., 2020; Racine et al., 2014). Tau has been revealed to be more than a stabilizer of microtubules, playing a role in a variety of biological processes, such as myelination, neurogenesis, motor function, learning, and memory (Fiock et al., 2020; Gold et al., 2014).

Abnormal tau protein has the property to aggregate with normal tau in endosomes, where they form fibrillar seeds. These fibrils are released from neurons, and spread pathology, resulting in an unstoppable neurodegenerative process in the brain of patients with AD (Asai et al., 2015). Even the process of tau protein hyperphosphorylation can also result in the degradation of axonal cytoskeleton proteins and subsequent loss of axon fibers (Michel et al., 2014).

Considering that tau is one of the main drug targets in AD, studies focusing on tau accumulation and dissemination modifiers are extremely important. Several tau‐positron emission tomography (PET) studies corroborate that tau pathology might spread through neuronal communication pathways, supporting the idea of trans‐synaptic transmission of misfolded tau in AD (Takeda et al., 2015; Vogel et al., 2020).

Furthermore, this process seems to be accelerated by regional Aβ pathology (Franzmeier et al., 2020; Takeda et al., 2015), and our individuals with aMCI have pathophysiological evidence of Aβ_42_ dysfunction. Longitudinal tau‐PET studies confirm that neocortical tau accumulation occurs almost exclusively in Aβ‐positive individuals (Jacobs et al., 2018; Pontecorvo et al., 2019). It is also possible that early Aβ pathology may trigger downstream events (e.g., spread of neocortical tau pathology), where the latter eventually becomes independent of the initiating event (Sanchez et al., 2021).

Besides, faster tau accumulation is associated not only with higher baseline tau and Aβ load but also with female sex and younger onset of AD (Karran et al., 2011). Although in our results we did not observe differences in terms of sex between the subjects, it is possible to notice a greater number of females in our study.

It is worth mentioning that our study did not investigate this accumulation of tau protein. We found relationships of CSF tau proteins levels with WM integrity measures, but only in MTL tracts. As already known, AD has been primarily considered a GM disease, with secondary WM disruption due to Wallerian degeneration (Knopman et al., 2018; Smith et al., 2020). In view of this, we chose to verify if there were correlations with the values of HV.

Through regression analysis, only HV remained a significant explanatory variable of CSF biomarkers. The HV better explained the variation found in the DTI measures (with weak to moderate effect sizes, explaining from 9% to 31%) than did CSF proteins. These findings provide evidence that hippocampal alterations remain a strong predictor of both tau deposition and WM changes.

We understand that the present study has some limitations. The absence of PET scan as an approach to analyze tau and amyloid could be a limitation, although CSF analyses can be very sensitive to detect AD pathophysiology. In fact, CSF alterations are, at least presently, the first alteration that can be observed in the disease process (Brueggen et al., 2019). The lack of information on AD biomarkers in the control group makes it difficult to understand if the proteins related to AD affect WM even before the appearance of any symptoms of the disease. However, our study presents patients with aMCI due to continuum AD, who show the pathological markers of the disease and can better help to understanding the progression of AD.

For DTI analyses, the multi‐atlas segmentation used in this study is a refined analytical tool able to access thinner and smaller tract that could be altered even in early stages of the disease. And, as already pointed out, it is a validated method that allows us to robustly analyze changes in early disease stages.

We also understand that the cross‐sectional nature and its correlational approach do not allow causal inference. For this, regression analysis could offer a better understanding of the hippocampal volume role in our results. It is noteworthy that there are few studies in Latin America involving such richness of details and the combination of different biomarkers. Therefore, our findings can help better clarify the neurodegenerative process in our population.

In conclusion, we found widespread alterations in WM integrity even in the earliest disease phase (MCI with altered amyloid). Also, HV better predicted MTL tracts integrity than did CSF biomarkers, what favors the hypothesis of WM disruption due to Wallerian degeneration.

## AUTHOR CONTRIBUTIONS

All authors made contributions to the conception, design, and analyses of the study. Raphael Fernandes Casseb contributed to the development of figures and supervision of statistics. Mateus Henrique Nogueira and Luciana Ramalho Pimentel‐Siva participated in the statistical analyses. Camila Vieira Ligo Teixeira and Ana Flávia Mac Knight Carletti participated in data collection. Helena Passarelli Giroud Joaquim, Leda Leme Talib, and Orestes Vicente Forlenza assisted in the analysis of CSF proteins. All authors have critically revised the manuscript for intellectual content and read and approved the final manuscript for submission.

## CONFLICT OF INTEREST

The authors declare no conflict of interest.

### ETHICS STATEMENT

The Medical Research Ethics Committee of the UNICAMP Hospital approved this study (CAAE: 09634412.5.0000.5404), and written informed consent (either from the subjects or from their responsible caretakers, if incapable) was obtained from all participants before the commencement of the study, in accordance with the Declaration of Helsinki.

### PEER REVIEW

The peer review history for this article is available at https://publons.com/publon/10.1002/brb3.2863

## Supporting information

Supp information

## Data Availability

The datasets used and/or analyzed during the current study are available from the corresponding author on reasonable request.
