# Supplementary material for: Whole‐brain DTI parameters associated with tau protein and hippocampal volume in Alzheimer's disease
Source: Brain Behav. 2023 Jan 4;13(2):e2863. doi: 10.1002/brb3.2863 (PMC9927845; doi:10.1002/brb3.2863)
Supplement: Supplementary file 1 — Supp information [file BRB3-13-e2863-s001.pdf]

Supplementary material

Table s1. List of white matter tracts obtained through analysis of Diffusion Tensor Imaging using MRICloud.

64 WM tracts - MRICloud
Cortico spinal Tract Left
Inferior Cerebellar Peduncle Left
Medial Lemniscus Left
Superior Cerebellar Peduncle Left
Cerebral Peduncle Left
Anterior Limb Internal Capsule Left
Posterior Limb Internal Capsule Left
Posterior Thalamic Radiation Left
Anterior Corona Radiata Left
Superior Corona Radiata Left
Posterior Corona Radiata Left
Cingulum Left
Cingulum Hippocampal Left
Fornix Stria Terminalis Left
Superior Longitudinal Fasciculus Left
Superior Fronto-occipital Fasciculus Left
Inferior Fronto-occipital Fasciculus Left
Sagittal Stratum Left
External Capsule Left
Uncinate Fasciculus Left
Pontine Crossing Tract Left
Middle Cerebellar Peduncle Left
Fornix Left
Genu Corpus Callosum Left
Body Corpus Callosum Left
Splenium Corpus Callosum Left
Retrolenticular Internal Capsule Left
Cortico spinal Tract Right
Inferior Cerebellar Peduncle Right
Medial Lemniscus Right
Superior Cerebellar Peduncle Right
Cerebral Peduncle Right
Anterior Limb Internal Capsule Right
Posterior Limb Internal Capsule Right
Posterior Thalamic Radiation Right
Anterior Corona Radiata Right
Superior Corona Radiata Right
Posterior Corona Radiata Right

**Cingulum Right**
**Cingulum Hippocampal Right**
**Fornix Stria Terminalis Right**
**Superior Longitudinal Fasciculus Right**
**Superior Fronto-occipital Fasciculus Right**
**Inferior Fronto-occipital Fasciculus Right**
**Sagittal Stratum Right**
**External Capsule Right**
**Uncinate Fasciculus Right**
**Pontine Crossing Tract Right**
**Middle Cerebellar Peduncle Right**
**Fornix Right**
**Genu Corpus Callosum Right**
**Body Corpus Callosum Right**
**Splenium Corpus Callosum Right**
**Retrolenticular Internal Capsule Right**
**Optic Tract Left**
**Optic Tract Righ**
**ICP-cerebellum Left**
**ICP-cerebellum Right**
**Cerebellum Branch-A Left**
**Cerebellum Branch-A Right**
**Cerebellum Branch-B Left**
**Cerebellum Branch-B Right**
**Tapatum Right**
**Tapatum Left**

Table s2. Pattern Matrix for Principal Components Analysis with Oblimin Rotation of Two Factor Solution of Diffusion Tensor Imaging parameters – White Matter tracts.

	Pattern coefficients	
	Component 1	***Component 2***
Fractional Anisotropy measure		
Body Corpus Callosum Left	**0.926**	
Body Corpus Callosum Right	**0.923**	
Genu Corpus Callosum Right	**0.902**	
Genu Corpus Callosum Left	**0.878**	-0.323
Splenium Corpus Callosum Right	**0.846**	
Splenium Corpus Callosum Left	**0.823**	
Fornix Right	**0.81**	
Anterior Corona Radiata Left	**0.785**	
Posterior Thalamic Radiation Right	**0.781**	
Anterior Corona Radiata Right	**0.764**	
Tapatum Right	**0.735**	
Cerebellum Branch-A Left		**0.744**
Cerebellum Branch-A Right		**0.73**
Axial Diffusivity measure		
External Capsule Right	**0.939**	
Superior Corona Radiata Right	**0.911**	
Superior Longitudinal Fasciculus Right	**0.891**	
Retrolenticular Internal Capsule Right	**0.861**	
Posterior Corona Radiata Right	**0.838**	
Anterior Limb Internal Capsule Right	**0.832**	-0.392
Anterior Corona Radiata Right	**0.816**	
Sagittal Stratum Right	**0.814**	
Posterior Limb Internal Capsule Right	**0.808**	
Inferior Fronto-occipital Fasciculus Right	**0.808**	
Body Corpus Callosum Right	**0.777**	
Cingulum Right	**0.747**	
Fornix Stria Terminalis Right	**0.711**	
Body Corpus Callosum Left	**0.705**	
Sagittal Stratum Left		**0.834**
Cingulum Hippocampal Left		**0.82**
Superior Longitudinal Fasciculus Left		**0.717**
Middle Cerebellar Peduncle Left		**0.715**
Retrolenticular Internal Capsule Left		**0.707**
Medial Diffusivity measure		
External Capsule Right	**0.931**	
Superior Corona Radiata Right	**0.907**	

Superior Longitudinal Fasciculus Right	**0.901**	
Cingulum Right	**0.882**	
Anterior Corona Radiata Right	**0.879**	
Inferior Fronto-occipital Fasciculus Right	**0.87**	
Retrolenticular Internal Capsule Right	**0.869**	
Sagittal Stratum Right	**0.853**	
Posterior Limb Internal Capsule Right	**0.847**	
Anterior Limb Internal Capsule Right	**0.824**	-0.349
Posterior Corona Radiata Right	**0.816**	
Superior Fronto-occipital Fasciculus Right	**0.79**	
Anterior Corona Radiata Left	**0.769**	
Posterior Thalamic Radiation Right	**0.719**	
Body Corpus Callosum Right	**0.715**	
External Capsule Left	**0.71**	
Middle Cerebellar Peduncle Left		**0.777**
Cingulum Hippocampal Left		**0.748**
Medial Lemniscus Right		**0.739**
Medial Lemniscus Left		**0.709**
Radial Diffusivity measure		
Anterior Corona Radiata Right	**0.911**	
Superior Longitudinal Fasciculus Right	**0.896**	
Cingulum Right	**0.89**	
External Capsule Right	**0.887**	
Superior Corona Radiata Right	**0.878**	
Sagittal Stratum Right	**0.844**	
Retrolenticular Internal Capsule Right	**0.842**	
Anterior Corona Radiata Left	**0.838**	
Inferior Fronto-occipital Fasciculus Right	**0.831**	
Cingulum Left	**0.806**	
Posterior Corona Radiata Right	**0.788**	
Superior Corona Radiata Left	**0.777**	
Posterior Thalamic Radiation Right	**0.769**	
Superior Fronto-occipital Fasciculus Right	**0.765**	
External Capsule Left	**0.756**	
Anterior Limb Internal Capsule Right	**0.746**	
Posterior Limb Internal Capsule Right	**0.739**	
Body Corpus Callosum Right	**0.716**	
Medial Lemniscus Right		**0.755**
Medial Lemniscus Left		**0.706**
Inferior Cerebellar Peduncle Right		**0.703**
Cerebellum Branch-B Left		**0.7**

Note: major loadings for each item are bolded

Table s3. Group comparison of WM integrity data in significant regions.

	Controls (n = 103)	aMCI (n = 44)	Mild AD (n = 29)
Fractional anisotropy			
**L Genu of CC**	0.57 (0.03)	0.56 (0.04)	0.54 (0.03) ^{a*}
**R Genu of CC**	0.56 (0.04)	0.55 (0.04)	0.53 (0.03) ^{a**}
**L Body of CC**	0.56 (0.03)	0.55 (0.03)	0.54 (0.02) ^{a*}
**R Body of CC**	0.56 (0.03)	0.55 (0.03)	0.53 (0.02) ^{a*}
**R Fornix**	0.51 (0.06)	0.48 (0.04)	0.43 (0.04) $^{a***, b**}$
**R Sagittal Stratum**	0.45 (0.01)	0.44 (0.02) ^{a*}	0.43 (0.01) ^{a***}
**L Sagittal Stratum**	0.45 (0.02)	0.44 (0.02)	0.43 (0.02) ^{a*}
**R Anterior Corona Radiata**	0.41 (0.02)	0.40 (0.02) ^{a*}	0.39 (0.02)
**L Posterior Thalamic Radiation**	0.48 (0.02)	0.47 (0.02) ^{a*}	0.46 (0.02) ^{a***}
**R Posterior Thalamic Radiation**	0.48 (0.02)	0.47 (0.02) ^{a**}	0.46 (0.02) ^{a***}
Mean diffusivity (x10^{-4})			
**R external capsule**	7.2 (0.4)	7.5 (0.6) ^{a*}	7.6 (0.6) ^{a*}
**R inferior fronto-occipital fasciculus**	7.6 (0.5)	7.8 (0.5)	8 (0.5) ^{a**}
**L Superior Corona Radiata**	7.1 (0.3)	7.2 (0.4)	7.5 (0.4) $^{a***, b*}$
**R Superior Corona Radiata**	7.3 (0.3)	7.6 (0.4) ^{a**}	7.7 (0.5) ^{a**}
**R superior longitudinal fasciculus**	7.4 (0.4)	7.6 (0.4) ^{a**}	7.8 (0.4) ^{a***}
**L Cingulum**	7.7 (0.3)	7.8 (0.3) ^{a*}	8.1 (0.3) $^{a***, b***}$
**R Cingulum**	7.6 (0.3)	7.8 (0.3) ^{a**}	8 (0.3) ^{a***}
**L Anterior Corona Radiata**	7.9 (0.4)	8.1 (0.5)	8.4 (0.5) $^{a***, b*}$
**R anterior corona radiata**	8 (0.4)	8.4 (0.5) ^{a**}	8.6 (0.5) ^{a***}
**R retrolenticular inetrnal capsule**	7.7 (0.4)	8.1 (0.5) ^{a**}	8.3 (0.4) ^{a***}
**R sagittal stratum**	8.1 (0.4)	8.4 (0.5) ^{a**}	8.8 (0.5) $^{a***, b*}$
**R posterior corona radiata**	7.6 (0.4)	8 (0.5) ^{a***}	8.1 (0.4) ^{a***}
**L Genu of CC**	9.7 (0.8)	10.1 (0.7)	10.6 (0.8) ^{a***}
**R Genu of CC**	7.2 (0.8)	7.5 (0.9)	7.6 (0.8) ^{a***}
**L Body of CC**	9.6 (0.7)	10 (0.8) ^{a**}	10.6 (0.6) $^{a***, b**}$
**R Body of CC**	9.5 (0.7)	10 (0.8) ^{a**}	10.5 (0.5) ^{a***}
**R Posterior Thalamic Radiation**	8.3 (0.4)	8.5 (0.5) ^{a**}	8.9 (0.6) $^{a***, b***}$
**R Superior Fronto-occipital Fasciculus**	7.8 (0.7)	8.1 (0.7)	8.4 (0.9) ^{a***}
**R Fornix Stria Terminalis**	9 (0.6)	9.2 (0.6)	9.9 (0.5) $^{a***, b***}$
**R Cingulum Hippocampa**	7.8 (0.3)	8 (0.3) ^{a**}	8.5 (0.3) $^{a***, b***}$
Axial diffusivity (x10^{-4})			
**L superior longitudinal fasciculus**	11.4 (0.5)	11.4 (0.5)	11.8 (0.4) $^{a**, b**}$
**R Sagittal Stratum**	12.3 (0.6)	12.6 (0.7) ^{a*}	13.1 (0.7) $^{a***, b*}$
**R external capsule**	10.5 (0.5)	10.9 (0.7) ^{a**}	11.1 (0.8) ^{a**}
**R superior corona radiata**	11.2 (0.5)	11.5 (0.5) ^{a**}	11.6 (0.5) ^{a**}
**L Superior Corona Radiata**	11 (0.4)	11.1 (0.4)	11.4 (0.5) $^{a***, b*}$
**R inferior fronto-occipital fasciculus**	11.8 (0.7)	12 (0.7)	12.4 (0.8) ^{a**}

**R superior longitudinal fasciculus**	10.9 (0.5)	11.3 (0.5) a**	11.5 (0.6) a***
**L Anterior Corona Radiata**	11.5 (0.4)	11.6 (0.6)	12 (0.5) a***, b**
**L Body of CC**	16.4 (0.9)	17 (0.1) a***	0.00177 (0.8) a***
**R Body of CC**	16.5 (0.9)	17.2 (1.1) a***	0.00177 (0.8) a***
**R Posterior Corona Radiata**	11.5 (0.6)	11.9 (0.6) a***	12.2 (0.6) a***
**R Anterior Corona Radiata**	11.7 (0.4)	12 (0.6) a***	12.4 (0.7) a***
**R Retrolenticular Internal Capsule**	12.4 (0.6)	12.9 (0.7) a***	13.2 (0.6) a***
**R Posterior Thalamic Radiation**	13 (0.6)	13.3 (0.6) a*	13.7 (0.7) a***, b**
**R Fornix Stria Terminalis**	13.9 (0.8)	14.2 (0.9) a***	15 (0.8) b***
**R Cingulum**	11.6 (0.4)	11.8 (0.5)	12.1 (0.5) a***, b*
**R Genu of CC**	17 (1.0)	17.6 (1.2)	18 (1.0) a***
**R Tapatum**	19 (1.7)	19.7 (1.7)	20 (1.6) a***, b*
**R Cingulum Hippocampal**	11.78 (0.5)	11.9 (0.4)	12.5 (0.5) a***, b***
Radial diffusivity ($x10^{-4}$)			
**L anterior corona radiata**	12.3 (0.7)	12.7 (0.9)	13.1 (0.9) a**
**R anterior corona radiata**	12.4 (0.8)	12.9 (1.0) a**	13.3 (1.0) a**
**L cingulum**	10.7 (0.5)	11.1 (0.6) a**	11.3 (0.6) a***
**R cingulum**	11.2 (0.5)	11.6 (0.6) a**	11.8 (0.6) a**
**R inferior fronto-occipital fasciculus**	11 (0.8)	11.3 (1.0)	11.8 (0.8) a**, b*
**R external capsule**	11.1 (0.8)	11.6 (1.1) a*	11.8 (1.1)
**R superior longitudinal fasciculus**	11.2 (0.7)	11.6 (0.8) a**	11.8 (0.8) a**
**L Superior Corona Radiata**	10.4 (0.6)	10.7 (0.8)	11.1 (0.8) a**
**R superior corona radiata**	10.7 (0.6)	11.2 (0.8) a**	11.4 (0.9) a**
**R sagittal stratum**	12 (0.7)	12.6 (0.9) a***	13.2 (0.9) a***, b***
**R retrolenticular internal capsule**	10.8 (0.7)	11.3 (0.8) a**	11.5 (0.7) a**
**R Posterior Corona Radiata**	11.3 (0.7)	11.8 (0.9) a***	12 (1.0) a***
**R Superior Fronto-occipital Fasciculus**	11.5 (1.2)	12.2 (1.4) a**	12.6 (1.6) a***
**R Body of CC**	12.1 (1.4)	12.9 (1.6) a**	13.9 (1.0) a***

Note: mean (standard deviation). Statistical analysis: MANCOVA with Bonferroni *post hoc* test. R: right; L: left; CC: Corpus Callosum.
a: different from controls
b: different from aMCI
* p <0.05
** p < 0.01
*** p < 0.001
